# Anti-Osteoporotic Effects of Antioxidant Peptides PIISVYWK and FSVVPSPK from *Mytilus edulis* on Ovariectomized Mice

**DOI:** 10.3390/antiox9090866

**Published:** 2020-09-15

**Authors:** Yunok Oh, Chang-Bum Ahn, Won Ho Cho, Na Young Yoon, Jae-Young Je

**Affiliations:** 1 Institute of Marine Life Sciences, Pukyong National University, Busan 48613, Korea; si565@daum.net; 2 Division of Food and Nutrition, Chonnam National University, Gwangju 61186, Korea; a321@jnu.ac.kr; 3 Department of Neurosurgery, Medical Research Institute, Pusan National University Hospital, Pusan National University School of Medicine, Busan 49241, Korea; mdcwh@naver.com; 4 Food & Safety Division, National Fisheries Research & Development Institute, Busan 46083, Korea; dbssud@korea.kr; 5 Department of Marine-Bio Convergence Science, Pukyong National University, Busan 48547, Korea

**Keywords:** osteoporosis, osteoblasts, alkaline phosphatase, bone mineral density, bioactive peptide

## Abstract

Numerous amounts of evidence suggest that bioactive peptides with diverse physiological activities can be nutraceuticals or potential drug candidates. In this study, blue mussel-derived antioxidant peptides PIISVYWK and FSVVPSPK were subjected to evaluate their osteogenic effect in mouse bone marrow mesenchymal stem cells (mBMMSCs) followed by an *in vivo* anti-osteoporotic effect. Treatment of PIISVYWK and FSVVPSPK on mBMMSCs stimulated alkaline phosphatase activity and calcification. Western blot results revealed that PIISVYWK and FSVVPSPK increased the expression of bone morphogenetic protein-2/4 (BMP-2/4) followed by upregulating p-Smad1/5, type I collagen, and transcription factors including Runx2 and osterix in mBMMSCs. Two peptides also activated the phosphorylation of MAPKs (p-p38, p-ERK, and p-JNK). Treatment of MAPK inhibitors significantly inhibited the BMP signaling pathway, indicating that PIISVYWK and FSVVPSPK stimulated osteoblast differentiation of mBMMSCs through the MAPK-dependent BMP signaling pathway. The anti-osteoporotic effect of PIISVYWK and FSVVPSPK in ovariectomized (OVX) mice was investigated. Treatment of PIISVYWK and FSVVPSPK for ten weeks showed a notable anti-osteoporotic effect in OVX mice via increasing bone mineral density and other bone parameters compared to OVX mice without peptides. Serum analysis also showed that treatment of PIISVYWK and FSVVPSPK completely reduced osteocalcin and ALP (alkAline phosphatase) activity. Taken together, these results suggest that PIISVYWK and FSVVPSPK could be health-promoting functional food ingredients against osteoporosis.

## 1. Introduction

There is a growing interest in the development of bioactive peptides (BAPs) with a potential application as an ingredient of functional foods and nutraceuticals as well as pharmaceuticals. Numerous amounts of evidence and awareness of health-promoting effects of BAPs have been accelerated to develop BAPs from food proteins by enzymatic hydrolysis, which is a well-known approach to produce BAPs without altering nutritional values. BAPs act as not only nutrients but also physiological modulating materials, and until now, antioxidant peptides with multifunctional activities are major BAPs developed from food proteins [1]. Recently, a number of marine-derived BAPs have been reported, and these BAPs have high potential nutraceutical and pharmaceutical values due to their bioactivities, including antioxidant, antimicrobial, antitumor, antihypertensive, and neuroprotective activities [2,3,4,5,6,7,8]. In our previous research, two octapeptides (PIISVYWK and FSVVPSPK) from blue mussel protein hydrolysates by enzymatic hydrolysis were identified and demonstrated their antioxidant and hepatoprotective effect [9]. Because antioxidant peptides are likely to possess a broad spectrum of bioactivities, it would be worthy investigating the health-promoting effect of utilizing antioxidant peptides.

Stem cells are special cells that are capable of differentiation into many different types of cells. Recently, potent BAPs that can modulate stem cell differentiation into osteoblast, which is involved in the bone formation, have been reported [10,11,12,13]. These BAPs stimulated osteoblast differentiation through activating BMP and/or Wnt/β-catenin signaling pathways, which are crucial pathways for osteoblast differentiation [1,2,3]. In addition, previous reports have shown that mitogen-activated protein kinase (MAPK) signaling is also involved in osteoblast differentiation [14,15]. These pathways finally stimulate expression of transcription factors, Runx2 and osterix, followed by activation of ALP and osteocalcin, which are the osteoblastic biomarkers, as well as mineralization. Thus, development of BAPs that can stimulate specific signaling pathway would be a good strategy for bone health against bone loss.

Osteoporosis is a common bone disease caused by imbalance between osteoblast and osteoclast activity. Currently, anticatabolic drugs such as bisphosphonates, calcitonin, and selective estrogen receptor modulators are mainly available in the market for osteoporosis treatment, but less anabolic agents are available [16]. Because of severe side-effects of anticatabolic drugs, it is urgent to develop agents that are safer and stimulate bone formation. The objective of this study is to identify an osteogenic effect with the molecular mechanism of blue mussel-derived antioxidant peptides PIISVYWK and FSVVPSPK and to evaluate the preventive effect on bone loss in ovariectomized (OVX) mice.

## 2. Materials and Methods

### 2.1. Materials

Cell culture media and buffer were obtained from HyClone (Waltham, MA, USA). All primary antibodies (BMP-2/4: Cat. No. sc-137087; p-Smad 1/5: Cat. No. sc-12353; Runx2: Cat. No. sc-390351; type 1 collagen: Cat. No. sc-8784; osterix: Cat. No. sc-393060; p-p38: Cat. No. sc-7973; p-ERK: Cat. No. sc-81492; p-JNK: Cat. No. sc-6254; β-actin: Cat. No. sc-8432), secondary antibodies, and MAPK inhibitors (SB203580, SP600125, and PD98059) were purchased from Santa Cruz Biotechnology (Santa Cruz, CA, USA). All other chemicals were of the highest commercial grade and purchased from Sigma-Aldrich (St. Louis, MO, USA).

### 2.2. Peptide Synthesis

Antioxidant peptides PIISVYWK and FSVVPSPK were chemically synthesized and supplied by Peptron, Inc. (Daejeon, Korea), and their purity (over 95%) was verified by HPLC-MS (Shimadzu LC-20A, Kyoto, Japan).

### 2.3. Cell Culture

Murine bone marrow mesenchymal stem cells (mBMMSCs, D1 ORL UVA, CRL-12424) were obtained from the American Type Culture Collection (ATCC, Manassan, VA, USA). The cells were maintained in Dulbecco’s Modified Eagle’s Medium (DMEM) supplemented with 10% fetal bovine serum (FBS) and 1% penicillin/streptomycin in a 5% CO_2_ humidified incubator at 37 °C.

To induce osteogenic differentiation, the cells were first grown to 80~90% confluence and cultured in an osteogenic differentiation medium (ODM, DMEM supplemented with 50 µg/mL ascorbic acid 10 mM β-glycerolphosphate and 10^−7^ M dexamethasone). ODM was changed every 2 days. The cells at passage 3 to 5 were used for osteogenic differentiation.

### 2.4. Qualitative Staining and Quantitative Assay of alkAline Phosphatase (ALP) Activity

mBMMSCs were seeded into a 96 well-plate at a density of 5 × 10^3^ cells per well and cultured in DMEM cell growth medium until confluence at 5% CO_2_ and 37 °C. For osteogenic differentiation, confluent cells were treated in ODM medium with various concentrations of two peptides for 3, 7, and 14 days. The medium was changed every 2 days. After 3, 7, and 14 days of exposure of two peptides, the cultured cells were carefully rinsed twice with PBS and fixed with 10% formalin for 5 min. After fixation, the cells were gently washed three times with PBS followed by addition of enough NBT/BCIP staining solution in the dark for 10 min at 37 °C. After removing the staining solution and washing with distilled water, ALP staining was visualized under a light microscope [10]. For quantitative ALP assay, the cells were lysed with sodium carbonate buffer (25 mM, pH 10) containing 0.1% triton X-100 after indicated time, following which lysates were scrapped off the plates and transferred into in 1.5 mL Eppendorf tubes. After centrifugation at 13,000 rpm at 4 °C for 15 min, the supernatant was collected and incubated with 25 mM sodium carbonate buffer containing 1.5 mM MgCl_2_ and 3.8 mM p-nitrophenyl phosphate at 37 °C for 90 min. After adding stop solution (0.2 M NaOH), ALP activity was calculated by measuring the optical absorbance at 405 nm using a microplate reader. 

### 2.5. Alizarin Red S Staining and Quantification of Calcification

mBMMSCs were seeded into a 96 well-plate at a density of 5 × 10^3^ cells per well and cultured in DMEM cell growth medium until confluence at 5% CO_2_ and 37 °C. Calcification was determined according to a previous method by Alizarin Red S staining (ARS) [11]. Briefly, the cells were incubated with ODM with two peptides for 21 days and then washed with PBS followed by fixation with 70% ethanol for 1 h at 4 °C. The fixed cells were stained with 2% ARS solution (pH 4.2) at room temperature for 15 min. Calcium deposits were immediately photographed under a light microscope. To quantify calcification, calcium deposits were extracted with 10% cetylprydiunium chloride in 10 mM sodium phosphate buffer (pH 7.0) for 20 min. Absorbance was measured at 562 nm.

### 2.6. Western Blot Analysis

mBMMSCs were cultured in a 60 mm cell culture dish until confluence at 5% CO_2_ and 37 °C. After confluence, the cells were differentiated in ODM medium containing 100 μM of two peptides for 7 days, and the medium was changed every 2 days. Whole cell lysates were prepared with a RIPA buffer containing protease and phosphatase inhibitors (Roche Applied Science, Branford, CT, USA), and protein concentrations of the lysates were determined using a colorimetric BCA protein assay (Pierce™ BCA Protein Assay Kit, Thermo Scientific^TM^, Boston, MA, USA). The proteins (20 μg) were separated on 10–12% SDS-PAGE and then transferred onto a PVDF membrane. The membrane was blocked in 1% of TBS-T buffer containing 5% skim milk for 1 h at room temperature and then incubated with primary antibodies (1:200 dilution) overnight at 4 °C. After washing with TBS-T three times, the membrane was incubated with Horseradish peroxidase-conjugated secondary antibody (1:1000 dilution) for 2 h at room temperature followed by another three times washing of TBS-T. The protein bands were visualized with an ECL kit (Pierce Biotechnology, Rockford, IL, USA) using a chemiluminescence imaging system (CAS 400SM, Davinchi-K, Seoul, Korea).

### 2.7. Animals and Experimental Design

Seven-week-old female C57BL/6 mice weighing 18 to 20 g were obtained from Envigo (Greenfield, IN, USA). The mice were housed at 23 ± 1 °C and fed commercial standard food pellets and water for 1 week to acclimatize before bilateral ovariectomies (OVX). The mice were recovered for 1 week after surgery and were randomly divided into the Sham mice group (PBS-treated), OVX mice group (PBS-treated), OVX-PIISVYWK mice group (50 μg of PIISVYWK/25 g mice/day), OVX-FSVVPSPK mice group (50 μg of FSVVPSPK/25 g mice/day), and OVX-Est mice group (0.5 μg of 17β-estradiol/25 g mouse/day) as a positive control. All mice were fed a low calcium pellet diet (0.01% calcium or less, Envigo, Indianapolis, IN, USA) to accelerate bone loss for 10 weeks. The normal mice group without surgery (PBS-treated) was fed a normal pellet diet. The mice were given 100 μL peptide (in PBS) or PBS by intraperitoneal injection. Body weights and food intakes were checked twice a week throughout the period of 10 weeks.

### 2.8. Ethic Statement

All experimental protocols and procedures in animals were approved by the animal care and use the committee of Pusan National University Hospital and performed in full accordance with the recommendations in the Guide for Pusan National University Hospital (PNUH-2017-108). All surgeries were performed under inhalant anesthesia, and all experiments were made to minimize the animals’ suffering ethically and humanely.

### 2.9. Analysis of Serum ALP and Osteocalcin (OCN)

The mice were fasted for at least 12 h, and blood samples were collected by cardiac puncture. The serum was recovered by centrifugation at 3000 rpm for 20 min at 4 °C. ALP activity and OCN concentration were determined using a SensoLyte pNPP ALP Assay kit (ANASPEC, Fremont, CA, USA) and Osteocalcin ELISA Kit (MyBioSource, San Diego, CA, USA) according to the manufacturer’s instructions.

### 2.10. Micro-CT Analysis

The femurs were dissected, and all tissues were removed followed by overnight fixation in a 4% formaldehyde. The bone architectures were evaluated using a 3D Micro-CT system (Inveon preclinical CT, Siemens Healthcare, Hoffman Estates, IL, USA) with an X-ray source at a voltage of 70 keV and a tube current of 400 μA. Two-dimensional micro-CT images of the femurs were scanned with an isotopic resolution of 20 μm voxel size and 2-D images in 80 continuous sections were stacked using Siemens Inveon Software for 3-D reconstruction. The bone morphologic parameters including bone mineral density (BMD), relative bone volume over total volume (BV/TV, %), trabecular number (Tb.N), trabecular separation (Tb.Sp), and cortical area thickness (Ct.Th) were calculated by the defined region of interest (ROI) of 3-D images, which was selected from a 0.1 to 2.5 mm area below the growth plate of each femur.

### 2.11. Statistical Analysis

All experiments were repeated at least three times, and the data were presented as the mean ± standard deviation (S.D). A two-tailed Student’s test was used for the comparison of two groups, and a one-way ANOVAs analysis was performed for multiple comparison tests to evaluate statistical significance. Values of * *p* < 0.05 and ** *p* < 0.01 were considered to indicate statistical significance.

## 3. Results

### 3.1. Blue Mussel-Derived Antioxidant Peptides PIISVYWK and FSVVPSPK Promote ALP Aactivity in BMMSCs

Since antioxidant peptides showed multifunctional bioactivity, in this study, as a part of our ongoing investigation on development of bone health-promoting biomaterials, we evaluated the osteogenic effect of two antioxidant peptides on mBMMSCs. 

**Figure 1:**
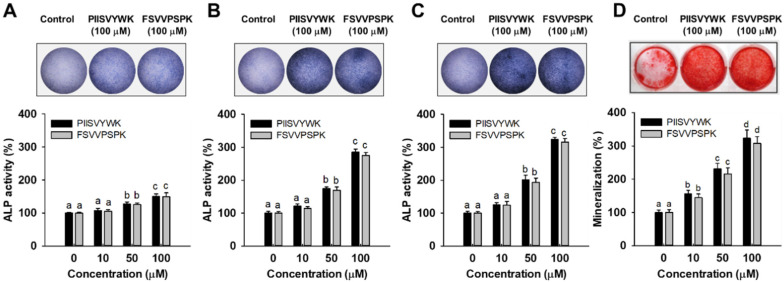
Effect of PIISVYWK and FSVVPSPK on ALP (alkAline Phosphatase) activity after incubating for (**A**) 3, (**B**) 7, and (**C**) 14 days and on (**D**) mineralization after incubating for 21 days in mBMMSCs (Murine bone marrow mesenchymal stem cells). Cells were treated with ODM (osteogenic differentiation medium) containing two peptides (0, 10, 50, and 100 μM) for the indicated incubation time. ODM was changed every 2 days. ALP staining and mineralization images are represented by the image at 100 μM. Results are shown as the mean ± S.D. with three determinations. ^a–d^ Bars with different letters indicate a significant difference at *p* < 0.05.

To evaluate the osteogenic effect of PIISVYWK and FSVVPSPK, ALP activity, an important marker of early stage osteogenesis in mBMMSCs, was first determined after incubation at 3, 7, and 14 days. Results of ALP activity from qualitative and quantitative analysis showed that two peptides stimulated ALP activity in mBMMSCs. At day 3, two peptides slightly stimulated ALP activity compared to the control group (without peptide treatment) (Figure 1A, lower panel). However, ALP activity was dramatically increased by 285 ± 9 (at 100 μM of PIISVYWK) and 275 ± 9% (at 100 μM of FSVVPSPK) after incubation for 7 days compared to the control group (Figure 1B, lower panel). At day 14, ALP activity was 324 ± 6 (at 100 μM of PIISVYWK) and 315 ± 11% (at 100 μM of FSVVPSPK) compared to the control group (Figure 1C). A rapid increase in ALP activity by PIISVYWK, and FSVVPSPK treatment was achieved at day 7, thereafter ALP activity was slightly increased. These results indicate that PIISVYWK and FSVVPSPK treatment stimulated ALP activity at an early stage during osteogenic differentiation of mBMMSCs. Representative ALP staining images are shown in the upper panel of Figure 1. The staining results are in agreement with quantitative analysis.

### 3.2. PIISVYWK and FSVVPSPK Promote Calcification in BMMSCs

To further evaluate the osteogenic effect of PIISVYWK and FSVVPSPK, calcium deposit, an indicator of late stage osteogenesis in mBMMSCs, was examined by ARS staining. As shown Figure 1D, the results of ARS staining showed that 100 μM PIISVYWK and FSVVPSPK treatment stimulated calcium deposits in mBMMSCs after incubation for 21 days (upper panel of Figure 1D). PIISVYWK and FSVVPSPK treatment significantly increased calcification in BMMSCs in a dose-dependent manner. The quantification results showed approximately 3.2- and 3.0-fold increments in calcium deposits by 100 μM PIISVYWK and FSVVPSPK treatment (lower panel of Figure 1D). 

### 3.3. PIISVYWK and FSVVPSPK Stimulate BMP and MAPK Signaling Pathways in BMMSCs

**Figure 2:**
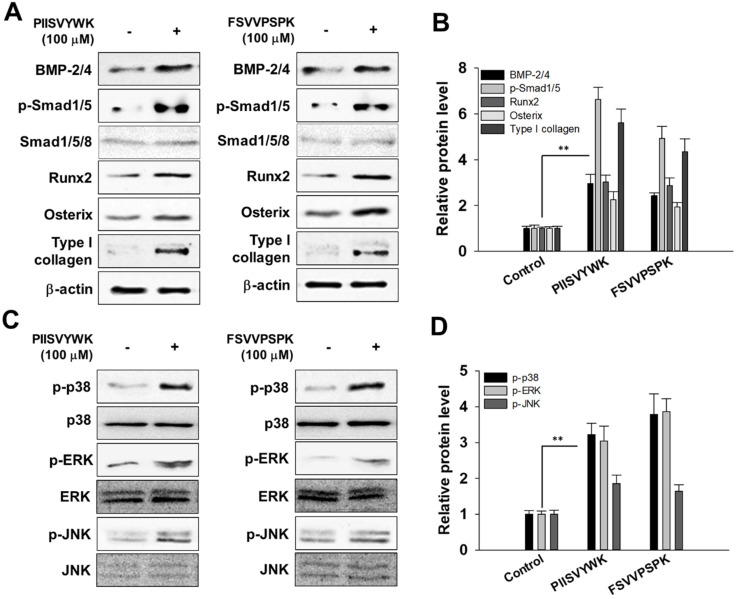
PIISVYWK and FSVVPSPK promote osteogenic differentiation of mBMMSCs (Murine bone marrow mesenchymal stem cells) through BMP (bone morphogenetic protein) and MAPK signaling pathways. (**A**) Western blot of BMP signaling, (**B**) quantification of BMP signaling, (**C**) Western blot of MAPK signaling, and (**D**) quantification of MAPK signaling. Cells were treated with ODM (osteogenic differentiation medium) containing two peptides (100 μM) for 7 days. ODM was changed every 2 days. Western blotting analysis was performed and analyzed band density by Image J software. The expression of each target protein was calculated as the relative expression to β-actin. Results are shown as the mean ± S.D. with three determinations. ** *p* < 0.01 vs. control group (0 μM of peptide).

In accordance with the osteogenic effect of PIISVYWK and FSVVPSPK, we investigated the potential mechanism underlying osteogenic differentiation induced by two peptides in mBMMSCs. We firstly investigated whether two peptides treatment could activate BMP signaling by Western blot analysis. As shown in Figure 2A,B, BMP-2/4 expression significantly increased after osteoinduction in the presence of two peptides for 7 days followed by phosphorylation of Smad1/5 and upregulation of transcription factors, including Runx2 and osterix. Type I collagen, the major protein synthesized by osteoblast, was also highly induced by PIISVYWK and FSVVPSPK. 

It is known that the MAPK signaling pathway is involved in the proliferation and differentiation of osteoblasts. Thus, to investigate the osteogenic effect of PIISVYWK and FSVVPSPK in mBMMSCs associated with MAPK signaling pathways, phosphorylated forms of p38, ERK, and JNK were assessed by Western blot analysis. As shown in Figure 2C,D, PIISVYWK and FSVVPSPK treatment activated phosphorylation of three MAPK molecules—p38, ERK, and JNK. These results indicate that differentiation of mBMMSCs into osteoblast by PIISVYWK and FSVVPSPK treatment was achieved through activation of BMP and MAPK signaling.

### 3.4. Effect of MAPK Inhibitors on PIISVYWK and FSVVPSPK-Mediated Osteogenic Differentiation

**Figure 3:**
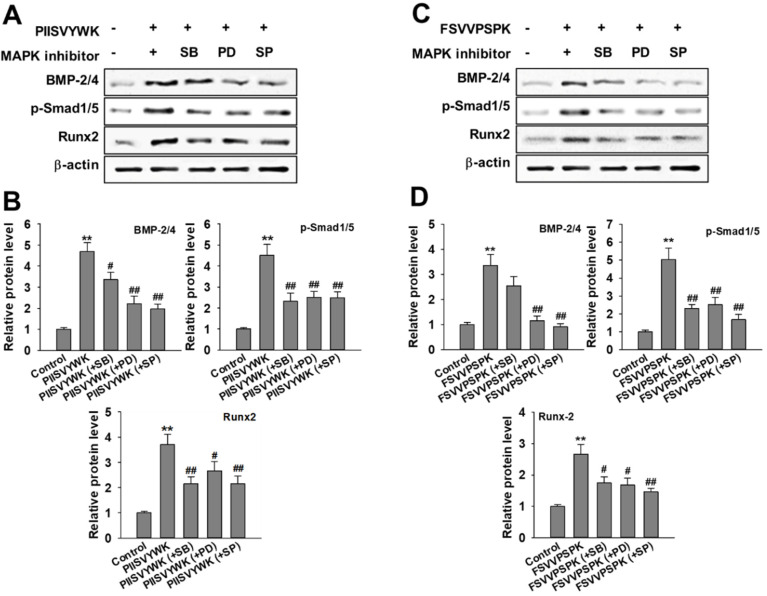
Effect of MAPK inhibitors on PIISVYWK and FSVVPSPK-mediated osteogenic differentiation (**A**–**D**). Cells were treated with three MAPK inhibitors including SB203580 (SB, 10 μM), PD98059 (PD, 20 μM), and SP600125 (SP, 10 μM) for 2 h prior to treatment with PIISVYWK and FSVVPSPK and incubated for 7 days. ODM (osteogenic differentiation medium) was changed every 2 days. Western blot analysis was performed and analyzed band density by Image J software. The expression of each target protein was calculated as the relative expression to β-actin. Results are shown as the mean ± S.D. with three determinations. ** *p* < 0.01 vs. control group (0 μM of peptide); ^#^ *p* < 0.05 and ^##^ *p* < 0.01 vs. peptide treatment group only.

**Figure 4:**
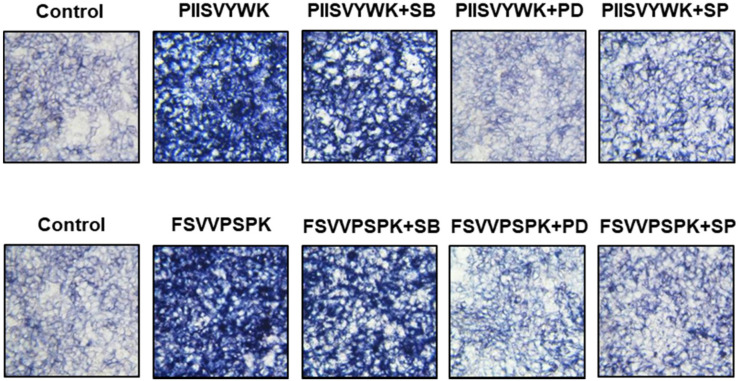
Effect of MAPK inhibitors on PIISVYWK and FSVVPSPK-induced ALP (alkAline Phosphatase) activity. Cells were treated with three MAPK inhibitors including SB203580 (SB, 10 μM), PD98059 (PD, 20 μM), and SP600125 (SP, 10 μM) for 2 h prior to treatment with PIISVYWK and FSVVPSPK and incubated for 7 days. ODM was changed every 2 days. The cells were staining with ALP staining reagents and observed under optical microscope (10× magnification).

Next, we evaluated the role of the phosphorylation of three MAPKs in osteogenic differentiation of BMMSCs in the presence of MAPK inhibitors. Cells were treated with MAPK inhibitors (p38 inhibitor SB203580, ERK inhibitor PD98057, and JNK inhibitor SP600125) prior to osteoinduction with PIISVYWK and FSVVPSPK for 7 days. Subsequently, changes in BMP signaling molecules including BMP-2/4, p-Smad1/5, and Runx2 were evaluated. As shown in Figure 3, the protein expression levels of BMP-2/4, p-Smad1/5, and Runx2 were markedly decreased in the cells treated with MAPK inhibitors compared to the control group (without MAPK inhibitors). BMP-2/4 expression was dominantly decreased in the presence of ERK and JNK inhibitors; however, downregulation of p-Smad1/5 and Runx2 was not significant in three MAPKs treatment. We further analyzed the role of three MAPK inhibitors by analyzing the ALP activity. As shown in Figure 4, two peptides treatment increased the ALP activity; however, cotreatment with three MAPK inhibitors decreased the ALP activity induced by treating two peptides. A weak role of p38 inhibitor was observed whereas ERK and JNK inhibitors showed a high abolishing effect in osteogenic differentiation induced by treating two peptides. Taken together, these results suggested that PIISVYWK and FSVVPSPK promote osteogenic differentiation of mBMMSCs through ERK- and JNK-dependent BMP signaling pathway.

### 3.5. PIISVYWK and FSVVPSPK Attenuate Bone Loss in OVX-Induced Osteoporosis Mice

**Figure 5:**
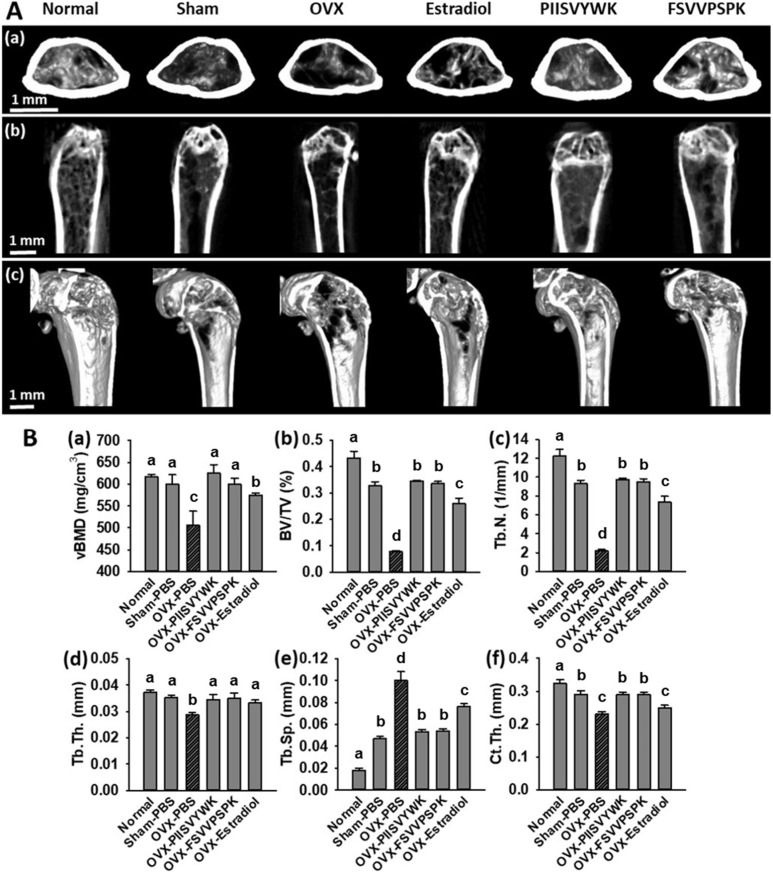
PIISVYWK and FSVVPSPK suppressed bone loss in OVX mice. (**A**) Micro-CT analysis of the distal femur region, (a) 2D images of cortical bone, (b) 2D images of distal femur, and (c) 3D reconstruction images of distal femur. (**B**) Analysis of micro-CT quantification of femoral bone parameters in the defined region of interest (ROI), (a) volumetric bone mineral density (vBMD), (b) trabecular bone volume/total volume (BV/TV), (c) trabecular number (Tb.N), (d) trabecular thickness (Tb.Th), (e) trabecular spacing (Tb.Sp), and (f) cortical wall thickness (Ct.Th) performed on the end of femur proximal spongiosa. All data are shown as the mean ± SD (*n* = 5). ^a–c^ Bars with different letters indicate significant difference (*p* < 0.05).

To explore the in vivo effects of PIISVYWK and FSVVPSPK, we measured bone mineral density (BMD) by a micro-CT scanner in OVX-induced osteoporotic mice. At the end of administration for 10 weeks, the body weights and food intakes of all mice were checked, and there were no significant differences among the experimental groups (data not shown). Representative 2-D and 3-D micro-CT images of femurs and the bone morphologic parameters are depicted in Figure 5. The OVX mice without peptide treatment showed significant bone loss and deterioration of bone structure compared to the sham and the normal mice. In particular, the femoral head of the OVX mice clearly showed a porous architecture (Figure 5A). Bone parameters of the OVX mice without peptide treatment showed a significant decrease in vBMD, BV/TV, Tb.N, Tb.Th, and Ct.Th and increase in Tb.Sp compared to the sham and normal mice (Figure 5B). The OVX mice treated with PIISVYWK and FSVVPSPK had higher vBMD, BV/TV, Tb.N, Tb.Th, and Ct.Th and lower Tb.Sp compared to the OVX mice without peptide treatment. The improved bone parameters by PIISVYWK and FSVVPSPK treatment were comparable to those of the sham mice and were higher than those of the 17β-estradiol-treated OVX mice. These results indicated that PIISVYWK and FSVVPSPK peptides showed a significant restoration of bone loss against OVX-induced osteoporotic mice.

### 3.6. PIISVYWK and FSVVPSPK Reduced OVX-Elevated Bone Turnover Biomarkers

**Figure 6:**
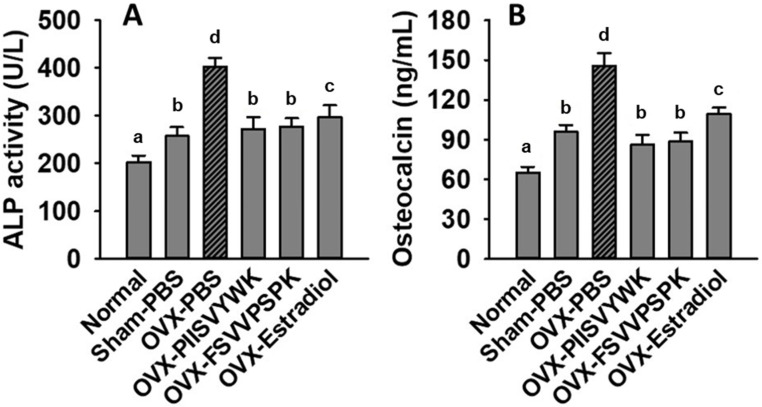
PIISVYWK and FSVVPSPK suppressed OVX-induced bone turnover. (**A**) Serum ALP activity and (**B**) serum osteocalcin. All data are shown as the mean ± SD (*n* = 5). ^a–d^ Bars with different letters indicate significant difference (*p* < 0.05).

To explore whether PIISVYWK and FSVVPSPK play a role in bone metabolism, we determined serum ALP and OCN concentration in OVX mice. As shown in Figure 6, the increase in serum ALP and OCN was found in the OVX mice without peptide treatment compared to the sham and normal mice, indicating high bone turnover. Surprisingly, PIISVYWK and FSVVPSPK treatment reversed these remarkable elevations in the OVX mice.

## 4. Discussion

The major goal of osteoporosis therapy is to restore bone mass through modulating the balance of bone formation and resorption. It can be performed by the use of antiresorptive agents and/or stimulating bone formation using anabolic agents. There have been attempts to develop anti-osteoporosis agents without side-effects. In recent years, several BAPs with a potential anti-osteoporotic effect have been developed from various endogenous proteins [12,17,18]. In addition, BAPs that could stimulate osteoblast differentiation and showed an anti-osteoporotic effect have also been developed from food proteins by enzymatic hydrolysis [10,11,19,20]. Therefore, in this study, we investigated whether blue mussel-derived antioxidant peptides PIISVYWK and FSVVPSPK could stimulate osteogenic differentiation of mBMMSCs.

ALP, an ecto-enzyme produced by osteoblasts, is an important marker in the early stage of osteoblast differentiation [21]. ALP is observed on the osteogenic cell surface and matrix vehicles, which is increased with commitment to osteoblast lineage [22]. Elevated extracellular phosphate, which is degraded by ALP, generates calcification in the matrix and mature osteoblasts. Thus, ALP is required for the development of calcification that is regulated in the late stage of osteoblast differentiation [23]. In the present study, ALP activity increased significantly in an early stage of differentiation on day 7 (Figure 1A–C), whereas the calcification process took a relatively long time on day 21 (Figure 1D). These results showed that PIISVYWK and FSVVPSPK peptides stimulated osteoblast differentiation of mBMMSCs through an association between ALP and calcification.

To elucidate the mechanism underlying the enhanced ALP activity and calcification, we investigated whether two peptides affect BMP signaling in osteoblast differentiation because BMPs are well known for their role in bone formation through the differentiation of MSCs into osteoblasts [24]. BMPs, a member of transforming growth factor, are known to be a crucial factor for regulating ostegenic differentiation through upregulating the Smad-dependent signaling pathway [25]. In this study, we demonstrated that PIISVYWK and FSVVPSPK treatment stimulated BMP signaling through the Smad-dependent pathway including the phosphorylation of Smad1/5 followed by activation of Runx2 and osterix, which lead to an increase in ALP activity and calcification in osteoblast differentiation. In addition, we examined whether two peptides could activate the MAPK pathway, which is also known to be involved in osteogenic differentiation [14,26], and found that two peptides activated MAPK phosphorylation during osteogenic differentiation of mBMMSCs.

To gain further insights into molecular mechanisms underlying two peptides-induced osteogenic differentiation, we evaluated the role of MAPK phosphorylation in osteogenic differentiation because recent advances revealed that MAPK phosphorylation plays a crucial role in osteogenic differentiation through modulating BMPs-Smad-dependent signaling [14,27,28]. Our results demonstrated that three MAPK inhibitors abolished the expression of BMP-2/4, phosphorylation of Smad1/5, and Runx2, which are activated by PIISVYWK and FSVVPSPK treatment. In addition, we also analyzed ALP activity in the presence of three MAPK inhibitors, and the results indicated that ERK and JNK inhibitors play a key role in osteogenic differentiation of mBMMSCs. Our results suggested that PIISVYWK and FSVVPSPK independently stimulated osteogenesis through the induction of JNK- and ERK-dependent BMP-2/4 expression and downstream signaling molecules such as phosphorylation of Smad1/5 and Runx2.

The OVX animal model has been widely accepted to study postmenopausal osteoporosis with high bone loss and turnover by estrogen insufficiency [29]. Deterioration of the micro-architecture in the trabecular bone in the OVX animal model was observed. We found that PIISVYWK and FSVVPSPK treatment protected bone loss and enhanced bone parameters in the OVX mice compared to the OVX mice without peptide treatment. These results suggested that blue mussel-derived antioxidant peptides PIISVYWK and FSVVPSPK were effective not only in maintaining bone loss but also in protecting bone microarchitecture in the OVX mice. We also analyzed biochemical markers of bone turnover in the OVX mice. ALP and OCN are secreted by osteoblasts and are essential for bone mineralization, formation, and turnover. An estrogen deficiency caused a high level of ALP and OCN in postmenopausal osteoporosis, indicating bone remodeling [30,31]. In this study, a high concentration of serum ALP and OCN in the OVX mice without peptide treatment compared to those in the Sham mice was observed. However, PIISVYWK and FSVVPSPK treatment significantly reversed these elevations in the OVX mice, indicating that PIISVYWK and FSVVPSPK protected OVX-induced bone loss through decreased bone turnover.

## 5. Conclusions

In conclusion, in vitro and in vivo findings indicated that antioxidant peptides PIISVYWK and FSVVPSPK from *M. edulis* may be helpful for bone health. Two peptides acted as an anabolic agent in vitro through activating MAPK-dependent BMP signaling and rescued bone loss in an estrogen-deficiency animal model. These findings provide a potential use of antioxidant peptides PIISVYWK and FSVVPSPK as a therapeutic drug for osteoporotic bone loss by estrogen deficiency.
